# A review on model-based and model-free approaches to control soft actuators and their potentials in colonoscopy

**DOI:** 10.3389/frobt.2023.1236706

**Published:** 2023-11-09

**Authors:** Motahareh Asgari, Ludovic Magerand, Luigi Manfredi

**Affiliations:** ^1^ Division of Imaging Science and Technology, School of Medicine, University of Dundee, Dundee, United Kingdom; ^2^ Division of Computing, School of Science and Engineering, University of Dundee, Dundee, United Kingdom

**Keywords:** endoscopic robot, soft medical robots, soft pneumatic actuator, control strategies, model-free control, model-based control

## Abstract

Colorectal cancer (CRC) is the third most common cancer worldwide and responsible for approximately 1 million deaths annually. Early screening is essential to increase the chances of survival, and it can also reduce the cost of treatments for healthcare centres. Colonoscopy is the gold standard for CRC screening and treatment, but it has several drawbacks, including difficulty in manoeuvring the device, patient discomfort, and high cost. Soft endorobots, small and compliant devices thatcan reduce the force exerted on the colonic wall, offer a potential solution to these issues. However, controlling these soft robots is challenging due to their deformable materials and the limitations of mathematical models. In this Review, we discuss model-free and model-based approaches for controlling soft robots that can potentially be applied to endorobots for colonoscopy. We highlight the importance of selecting appropriate control methods based on various parameters, such as sensor and actuator solutions. This review aims to contribute to the development of smart control strategies for soft endorobots that can enhance the effectiveness and safety of robotics in colonoscopy. These strategies can be defined based on the available information about the robot and surrounding environment, control demands, mechanical design impact and characterization data based on calibration.

## 1 Introduction

Colorectal cancer (CRC) is the third most common cancer worldwide and responsible for approximately 1 million deaths annually. Early screening is essential to increase the chances of survival, and it can also reduce the cost of treatments for healthcare centers. There are several methods for colon cancer screening, including non-invasive and invasive methods. Non-invasive methods include the faecal occult blood test (FOBT), faecal immunochemical test (FIT), liquid biopsy, and wireless colon capsule (Romero-Vázquez et al., 2014). Invasive methods include colonoscopy, sigmoidoscopy, etc., of which colonoscopy is considered the gold standard for CRC screening and treatment (Nee et al., 2020).

However, colonoscopy has several drawbacks, including difficulty in manoeuvring the device, patient discomfort, and high cost. Soft endorobots, small and compliant devices that can reduce the force exerted on the colonic wall, offer a potential solution to these issues. However, controlling these soft robots is challenging due to their deformable materials and the limitations of mathematical models.

In this Review, we discuss model-free and model-based approaches for controlling soft robots that can potentially be applied to endorobots for colonoscopy. We highlight the importance of selecting appropriate control methods based on various parameters, such as sensor and actuator solutions. This review aims to contribute to the development of smart control strategies for soft endorobots that can enhance the effectiveness and safety of robotics in colonoscopy.

### 1.1 Wireless colon capsule (WCC)

Wireless Colon Capsule (WCC) is a non-invasive method for screening the colon that was introduced by Given Imaging Ltd. in 2006. The first-generation WCC, called PillCam COLON, is a capsule that is swallowed by patients and has two cameras at both tips. An external data system connected to the patient is used for recording the video streamed by the capsule (Iddan et al., 2000). However, this method has several downsides. First, it is an expensive method. Secondly, it requires more colon preparation compared to conventional colonoscopy (Spada et al., 2012). Thirdly, it is challenging to control the WCC for inspecting a certain part of the colon. Fourthly, this procedure is time-consuming, requiring extra time to analyse the video (Van Gossum et al., 2009; Thygesen et al., 2019). Moreover, these WCCs are not able to remove polyps or perform biopsies (Martin et al., 2020). Overall, while WCC offers a non-invasive alternative to colonoscopy, it has several limitations that may make it less suitable for some patients. The inability to remove polyps or perform biopsies and the time-consuming nature of the procedure may limit its diagnostic capabilities. Furthermore, the cost and the need for extensive colon preparation may make it less practical in some healthcare settings.

### 1.2 Colonoscopy

**FIGURE 1:**
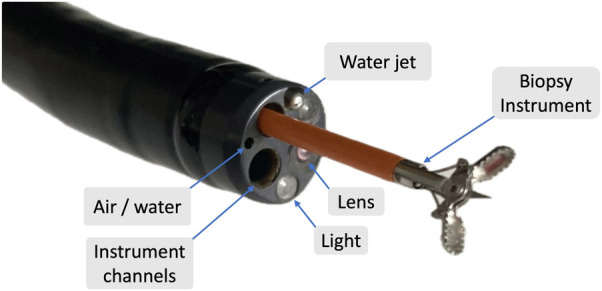
Colonoscope tip.

Colonoscopy is an invasive method used for screening the colon. This procedure enables the examination of the entire colonic mucosa and removal of polyps (Winawer et al., 1993). Polyps are cellular growths that can develop on the colon’s surface and may have the potential to turn into cancer (Winawer, 1999; Granados-Romero et al., 2017). Removal of polyps or polypectomy plays a crucial role in reducing mortality rates (Winawer et al., 1993). During a colonoscopy, a tube called a colonoscope, which measures 1.6 m in length and up to 15 mm in external diameter, is used to visualize the colon. The instrument is equipped with tools for observing and insufflating the colon, as well as one or two channels for biopsy and suction. To ensure unobstructed visualization, any residual stool is removed via water, and CO2 is used to expand and stretch the lumen (Manfredi, 2021). The colonoscopy tip is depicted in Figure 1. Preparation of the colon is critical before the procedure. Patients should adhere to a special diet, use laxatives, and consume 2 L of solution 24 h before the examination to clean out the colon and prepare it for the procedure (Sharma et al., 2020). Poor colon preparation can limit clinical output and, in some cases, even cause the procedure to be aborted (Brahmania et al., 2012). During the procedure, patients are positioned on their side, and the colonoscope is inserted through the anus, rectum, and sigmoid colon until it reaches the cecum. Navigation through the mobile part of the sigmoid colon is challenging and often the most painful part of the procedure (Shah et al., 2002). The procedure may take over 40 min to complete (Jain et al., 2016). Colonoscopy is typically performed in two phases. In the first phase, the main goal is to reach the cecum, which takes about 20 min. In the second phase, the colonoscope is removed while inspecting the colonic wall for any abnormalities (Jain et al., 2016). A snare is used to remove polyps for tissue analysis, i.e., biopsy. Tattoos are used to mark the area for any required colonic resection.

However, colonoscopy has some limitations that should be considered. Firstly, bowel preparation is necessary, which can be challenging and not well-accepted by patients (Kelly et al., 2012). Poor colon preparation can increase the risk of missing polyps and interval cancers (Kim, 2012). Secondly, this procedure can cause discomfort and pain for patients, leading to their reluctance to undergo the procedure (Holme and Bretthauer, 2016). In fact, while intestinal insufflation plays a crucial role in enhancing colonoscopy visualization, it is also one of the primary reasons for patient discomfort and pain. It is worth mentioning that patients’ discomfort can result not only from physical sensations but also from emotional factors, such as embarrassment or anxiety about the procedure. Moreover, pain and discomfort can be influenced by other factors, including the patient’s sex, body mass index, age, and the level of experience of the colonoscopist. Rare cases of colonic wall damage have been reported (Morris et al., 2008). Additionally, colonoscopy requires long time for training (Siau et al., 2019), and the years of experience and training of clinicians have an impact on the success of the procedure (Lee et al., 2014; Siau et al., 2018). Moreover, the use of sedation or anesthesia to reduce pain and discomfort can increase the cost of the procedure and the recovery time for patients, and the risk of complications (Cooper et al., 2013; Wernli et al., 2016). The average cost of a colonoscopy provided by the NHS in the United Kingdom is £624. Disease transmission has also been reported as a potential issue in colonoscopy (Kenters et al., 2015; Wang et al., 2018b), which can be prevented by using disposable equipment (Ciocîrlan, 2019). Due to the increasing number of people requiring colonoscopy every year, the waiting list for patients is getting longer due to a limited workforce (Young et al., 2019). Therefore, there is a need for alternative methods for screening the colon.

## 2 Endorobots for colonoscopy

Soft endorobots, small robots being made form soft materials for endoscopic applications, also have the potential to improve the training process for clinicians since they provide a more controlled and repeatable environment for training compared to the current method of learning through live patients. Additionally, endorobots can be equipped with advanced imaging technologies, such as fluorescence imaging or virtual reality, to enhance the accuracy of polyp detection and localization. However, there are still some challenges to be addressed before endorobots can be widely used for colonoscopy. One of the main issues is the design of the robot’s locomotion system, which should be able to navigate through the complex and tortuous environment of the colon while minimizing the risk of injury to the colon wall. Another challenge is the development of a precise and responsive control system for the robot’s tip to enable effective surgical procedures while minimizing the risk of damage to the colon wall. Additionally, the cost and maintenance of the endorobots may be a concern, especially for smaller clinics or hospitals with limited resources (Manfredi, 2021). In conclusion, utilizing endorobots for colonoscopy has the potential to overcome the limitations of the current procedure and improve the accuracy and safety of colon screening. However, more research and development are needed to address the challenges and make endorobots a practical and accessible solution for colonoscopy.

There are some challenges in designing these endorobots as follows (Manfredi, 2021):

1) There is a restricted space for placing the essential instruments.
2) The surface of the colon is slippery.
3) The shape of the colon is long and tortuous.

One potential challenge for using endorobots for colonoscopy is ensuring that the robot is equipped with the necessary capabilities to perform a thorough examination of the colon within a reasonable amount of time. The robot should be designed to have a similar diameter to the existing colonoscope (12–15 mm), with two channels for operation, and it should not exceed a diameter of 20 mm. Additionally, the robot must be equipped with a high-definition camera that provides high-quality video streaming for accurate screening. An interventional instrument is also necessary to remove polyps and perform biopsy. To enhance visibility during the procedure, gas insufflation should be used to expand the lumen, and a waterjet is used to remove any residual stool inside the colon (Manfredi, 2021).

To enable the functionalities of the endorobot discussed earlier, a tether to an external control panel is necessary. However, certain factors, such as the number of wires used to comprise the tether, the tether’s outside diameter, the robot’s weight, and the friction against the colon mucosa, can affect the production of drag force (Alcaide et al., 2021). Overcoming this force is crucial for the locomotion system to move the endorobot forward (Alcaide et al., 2021). Thus, the design of the endorobot must take into consideration the drag force to enable efficient movement inside the colon.

Beyond design considerations, there remain significant challenges in the realm of control. These challenges stem from the robot’s numerous degrees of freedom, nonlinear behavior, and hysteresis, making it difficult to establish a precise mathematical model and control it effectively. Additionally, the soft materials employed in the robot can introduce delays in response time, making real-time feedback for closing the control loop a complex endeavor. While the flexibility of these robots can reduce the applied force on the colonic wall and lower the risk of damaging the colon, achieving sufficient stiffness for tasks such as reaching specific movements or maintaining stability while collecting tissue samples presents another formidable challenge. From a mechanical point of view, reproducing these robots with the same characteristic properties, performance, and durability can be demanding.

### 2.1 Activation and power systems

The design of the activation system for endorobots presents various challenges, including generating sufficient force to overcome tether drag, achieving an appropriate locomotion speed, reducing the applied force to minimize patient discomfort, and ensuring precise control for surgical tasks (Manfredi, 2021). Activation systems can be categorized based on their design, with external, wireless, and internal options available. External activation relies on powerful and heavy external actuators connected to the main device via mechanical transmission such as cables and pulleys, which can increase force at the distal part while keeping the internal part small (Manfredi, 2021). However, cable transmission can generate friction force, reducing output force (Agrawal et al., 2010) and increasing the stiffness of the tether. Alternatively, a wireless activation system utilizing a magnetic field has been proposed, with the options of a small permanent magnet inside the mini robot and an external magnetic field outside the colon (Manfredi, 2021).

Martin et al. (2020) utilized an internal and external magnets for a wireless activation system. It is important to note that the external permanent magnet has a limited volume compared to the coil, but it can generate force at a greater distance. In other words, the use of permanent magnets, such as neodymium magnets, can increase the force exerted due to their strong magnetic field despite their compact size. However, using the coil can enhance the controllability of the target device (Edelmann et al., 2018). This system eliminates the need for a cable, which addresses some of the disadvantages of the external activation system, such as tether drag and increased friction. However, magnetic field interference can be a significant issue. Although some endoscopy procedures have utilized wireless methods, the internal activation system is another option. However, internal locomotion of the capsule endoscope has some drawbacks, such as the need for high power, unstable movement, and the impossibility of integrating high-tech technology into a small capsule (Liu et al., 2015). Since the actuators used for internal activation are small in size, their actuation process is limited, resulting in restricted output force/torque. To address this, mechanical solutions like a gearbox can be used (Manfredi, 2021). However, the complexity of the design makes it difficult to reduce costs (Martin et al., 2020). Therefore, the selection of an activation system depends on various factors, such as design, required force, and available space, which must be taken into account beforehand.

When it comes to functionality, traditional robots differ significantly from biological systems. Animals’ bodies are characterized by greater redundancy, imprecision, and weakness compared to rigid robots, yet they can perform a multitude of tasks with remarkable effectiveness (Della Santina et al., 2021) due to the elasticity and compliance of their muscle-skeletal system (Della Santina et al., 2020). In many studies of biomechanics, researchers have found that incorporating elastic elements into the rigid bodies of robots can enhance their performance and make them more comparable to natural species, resulting in the development of soft robots (Della Santina et al., 2020). Soft robot can be comprised of soft actuators and/or soft sensors.

### 2.2 Sensing and actuating

Soft medical robots can be described as electro-mechanical devices that are soft, flexible and capable of performing various medical tasks. To effectively execute their functions, these robots must be able to adjust their stiffness. In minimally invasive surgery, there is a critical demand for not only appropriate deformation, navigation and interaction with soft human organs, but also the appropriate stiffness required for tasks such as tumour removal (Zhang and Lu, 2020). Therefore, the design and structure of the sensing and actuating system of these robots play a significant role in the achieving proper task performance.

The sensing components of soft medical robots need to be sufficiently flexible, durable, and expandable to ensure they do not hinder or significantly alter their capabilities (Zhang and Lu, 2020). The type of the sensor used in the medical robots depends on several parameters. From the control perspective, the critical factor in sensor selection is identifying the element that needs to be monitored for feedback in the control loop.

Soft sensors are developed by integrating electronically conductive fillers into soft actuators. There are different types of electronically conductive fillers that can be used for this purpose, such as liquid conductors, nanomaterials, and conductive fabrics (Kim et al., 2021). To detect the deformation of a soft robot, such as strain, curvature, and compression, the electrical changes of the fillers, such as resistance (Shih et al., 2019) or capacitance (Li et al., 2016), are measured.

Soft sensors have been integrated with various robotics technologies, such as actuators, grippers, manipulators, and wearable gadgets, to gain a better understanding of physical interactions between soft sensors and their surroundings or their own physical states. When incorporated into or attached to grippers, soft sensors can measure the magnitudes and positions of objects touched, predict the sorts of materials and forms of grasped objects, and recognize object slippage by assessing contact information obtained from post-processed sensor data. Array-type soft pressure sensors in soft mobile robots or manipulators can estimate the locations and movements of the robotic systems, as well as the distribution of contact force during interaction. Soft wearable devices equipped with soft sensors can assess human body movement, such as hand or leg motions and gait, due to their softness and elasticity features. However, due to the hyper-elastic material of the soft sensors, their physical behaviour is unpredictable and complicated, leading to nonlinearity, hysteresis, creep, and drift, which also impact their electrical response (Kim et al., 2021).

The power needed to perform flexible functions and predicted tasks is supplied by flexible actuation. Consequently, for soft medical robots, it is crucial to develop flexible actuation systems capable of generating substantial forces (Zhang and Lu, 2020). To create soft actuators capable of converting various energy inputs into mechanical energy outputs, it is important to consider material selection, structural design, and manufacturing methods (Li et al., 2022).

Soft actuators can be classified into four groups based on their actuation mechanisms, which include: pneumatic actuators (Walker et al., 2020), cable-driven actuators (Calisti et al., 2011), Electroactive Polymers (EAPs) (Bar-Cohen, 2000), and Shape Memory Alloys (SMAs) (Koh and Cho, 2012). Soft pneumatic actuators (SPAs) are widely used in robotics due to their flexibility in motion. However, the use of traditional sensors in SPAs can limit their movement. Soft sensors integrated into SPAs can provide some information such as contact force, pressure or bending motion. This has led to the frequent use of soft sensors in this area (Kim et al., 2021).

## 3 Control strategies

In recent years, there has been an evolution in the control strategies due to three significant needs: 1) to cope with complicated systems, 2) to meet demanding design requirements, and 3) less accurate information about the systems and environments. This has led to a re-evaluation of traditional control methods and the generalization of control concepts (Antsaklis, 1990). Soft robots, as a new area of robotics, present new challenges in terms of control strategies.

Model-based methods are typically the first approach used to control many systems, with data-driven and machine learning algorithms being used when stronger control is required. In the field of rigid robots, control methods have evolved from the frequency domain, to linear state space control, to the fully nonlinear area, and most recently, machine learning approaches. However, in the field of soft robotics, machine learning techniques were initially used to control them, except in fully kinematic quasi-static scenarios (Della Santina et al., 2021). There was a belief for many years that model-based methods could not be feasible for controlling soft robots. However, this view has changed due to two major factors. Firstly, it has been proven theoretically and experimentally that if the dynamics of the soft robot are estimated approximately, the feedback strategy is robust. Secondly, newly emerged techniques for modeling the soft robots, named “finite-dimensional,” can be accurate (Della Santina et al., 2021). There are many factors that influence the control system of soft robots, such as intrinsic dynamics, elastic potential field, and different ways for sensing and actuating. All of these factors should be considered, making the control part challenging (Della Santina et al., 2021). Despite the existence of many papers on soft robots, only a few have been focused on control strategies (George Thuruthel et al., 2018), with this paper mainly focusing on model-free methods.

### 3.1 Model-based control

Model-based control of soft robots is challenging due to the infinite dimensions of their state space which makes it difficult to define the mathematical model of the robot (Trivedi et al., 2008). To simplify the model, Piecewise Constant Curvature (PCC), a decreased kinematic model is commonly used in controlling soft robots (Webster and Jones, 2010). In a static situation with limited interaction with the environment, kinematic methods along with low-level high gain feedback can be used to control soft robots. However, in a dynamic situation where dynamic tasks are required and the robot interacts constantly with the environment, a dynamic model of the soft robot is necessary (Della Santina et al., 2018).

#### 3.1.1 Piecewise constant curvature

**FIGURE 2:**
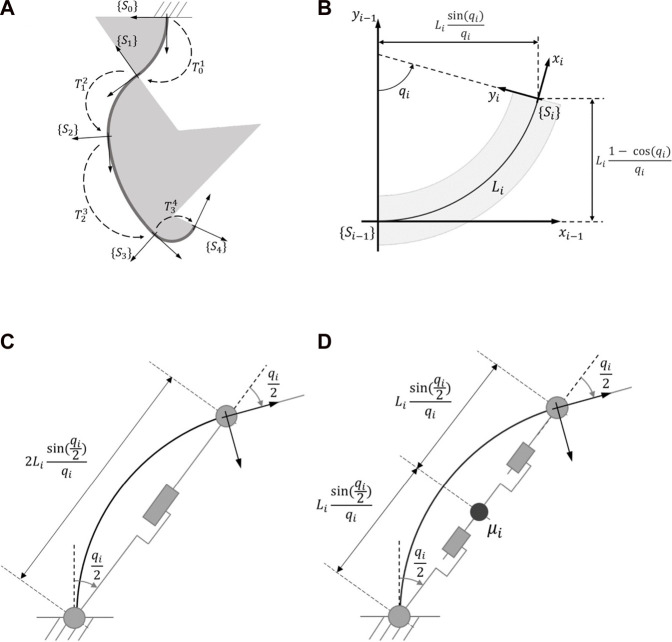
**(A)** A robot with 4 piecewise constant curvature elements, $\left\{s_{0}\right\}$ is a robots’ base frame and $\left\{s_{n}\right\}$ is a reference frame **(B)** kinematic model of the *i*th planar constant curvature segment, $q_{i}$ and $L_{i}$ representing the degree of curvature and length of a segment respectively **(C)** an examples of augmented robot RPR **(D)** an examples of augmented robot RPPR which considers the mass in the middle (Della Santina et al., 2018).

Della Santina et al. proposed a control method based on an “augmented formulation” that connects a soft robot to a rigid serial manipulator composed of a parallel elastic mechanism. The method uses controlling methods for rigid robots but requires a complete match between the soft robot and a rigid robot based on the assumption of piecewise constant curvature (PCC) (Della Santina et al., 2018). The kinematics of the soft robot are obtained based on certain hypotheses, including considering the soft robot, which has infinite dimensions, as a robot consisting of fixed sections with a constant curvature (CC) in a way that each curve is distinctive, with n CC sections and a set of reference systems $\left\{s_{0}\right\}\backslash \ldots \left\{s_{n}\right\}$ connected at the tip of each segment and the configuration of each segment can be represented by a single variable (Figure 2A). The kinematics of the soft robot can be defined as n homogeneous transformations $T_{0}^{1},\ldots ,T_{n-1}^{n}$ , with $q_{i}$ as the degree of curvature and $L_{i}$ as the length of a segment (Figure 2B) (Della Santina et al., 2018).

$$T_{i-1}^{i}=\left[\begin{array}{ccc} \cos\left(q_{i}\right) & -\sin\left(q_{i}\right) & L_{i}\frac{\sin\left(q_{i}\right)}{q_{i}}\\ \sin\left(q_{i}\right) & \cos\left(q_{i}\right) & L_{i}\frac{1-\cos\left(q_{i}\right)}{q_{i}}\\ 0 & 0 & 1 \end{array}\right]$$ (1)

In summary, the augmented formulation proposed by Della Santina et al. uses the assumption of piecewise constant curvature (PCC) to model the soft robot as a rigid serial manipulator with parallel elastic mechanisms. Each CC segment of the soft robot can be matched to a PRP^1^ robot (Figure 2C), and the state space of the rigid robot can be used to represent the augmented state of the PCC soft robot. A mapping function *ξ* = m(q) is used to relate the joint numbers for each CC section, and the tip point of each CC section should coincide with the corresponding reference point of the rigid robot. The soft robot and augmented robot should have the same inertial properties, and a proximate mass distribution is considered for each CC section. As a result, a PCC soft robot can be dynamically represented as a constant rigid robot called RPPR (Figure 2D).

$$B\ddot{q}+\left(C+D\right)\dot{q}+G_{G}\left(q\right)+K_{q}=\tau +J^{T}f_{ext}$$ (2)

The dynamic model for the soft robot can be expressed as Eq. 2 where B, C, G, D, and K represent the inertia, Coriolis and centrifugal term, effect of gravity, damping, and stiffness, respectively. The control term and external wrenches are represented by *t* and $f_{ext}$ in order. This model can be used for model-based control methods after obtaining the model of the soft robot using the proposed technique. However, this method has some disadvantages, such as uncertainties due to considered hypotheses like mass distribution and the limitation of the constant curvature assumption for some soft robots. In situations where the system is not well-known, traditional model-based control approaches may fail (Ijspeert, 2008). In addition to model-based and model-free control methods, other features like high compliance, damping, and elasticity should also be considered in controlling soft robots. The next section will discuss the impact of feedback and feedforward control on these features.

### 3.2 Feedback and feedforward control

**FIGURE 3:**
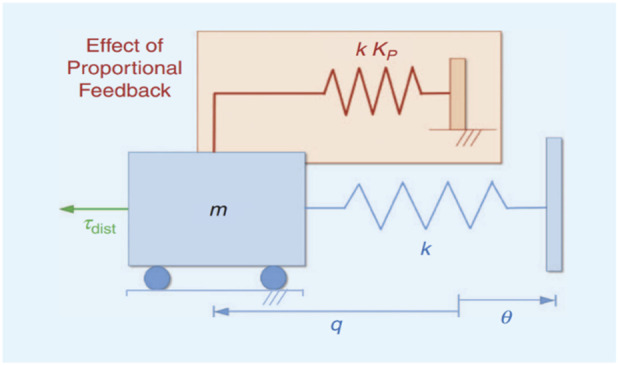
The effect of proportional feedback on a simple soft mechanism (Della Santina et al., 2017) ©2017 IEEE.

Della Santina et al. investigated the impact of feedback and feedforward control on a simple soft mechanism consisting of a mass and spring (Della Santina et al., 2017). They found that adding feedback/reactive control to the system increased the natural stiffness and damping, akin to adding a parallel spring, as demonstrated in Figure 3. The dynamic model of the system was given by Eq. 3, where β , k, and $\tau_{dis}$ represent physical damping, stiffness, and non-modeled dynamics, respectively. To compensate for $\tau_{dis}$ and regulate motion q, the control policy given by Eq. 4 was applied. Substituting (4) into Eq. 3 resulted in a new dynamic model, Eq. 5, which showed that adding feedback control increased the natural damping and stiffness parameters by $\left(1+\frac{k}{\beta }k_{d}\right)$ and $\left(1+k_{p}\right)$ respectively. Applying this approach to the stiffness of soft robots, it has been shown that using feedback control to achieve suitable tracking performance comes at the cost of reducing compliance. On the other hand, feedforward/anticipatory control can reduce the demand for high-gain feedback control to achieve optimal control without affecting the softness of the robot. However, the success of feedforward control relies on having an accurate model of the system, which is often difficult to obtain. Machine learning algorithms can be used to overcome this limitation.

Therefore, a combination of feedback and feedforward control can be used to achieve optimal control of soft robots while maintaining their softness.

$$m\ddot{q}+\beta \dot{q}+kq=k\theta +\tau_{dis}$$ (3)

$$\theta =-k_{p}q-k_{d}\dot{q}$$ (4)

$$m\ddot{q}+\beta \left(1+\frac{k}{\beta }k_{d}\right)\dot{q}+k\left(1+k_{p}\right)q=\tau_{dis}$$ (5)

### 3.3 Model-free control

Compared to rigid robots, soft robots possess desirable characteristics such as flexibility, deformability, and adaptability. However, these traits can introduce nonlinearity and hysteresis, making it challenging to model, calibrate, and control soft robots (Amjadi et al., 2016; Kim et al., 2021). Nonlinearity implies that the relationship between the input and output of the system is not linear, while hysteresis suggests that the system’s behaviour is time-dependent. Additionally, other factors such as creep, drift, and a high degree of freedom can exacerbate the hysteresis and lead to complex behaviour, further complicating the mathematically modeling process (Kim et al., 2021). As a result, conventional control methods may not be suitable for soft robots (Thuruthel et al., 2019). Various model-free approaches have been explored to control soft robots, including machine learning algorithms, neural networks, and fuzzy control. Machine learning algorithms have shown remarkable performance in addressing nonlinear problems in various fields (Weinberger et al., 2004; Yu et al., 2009) and have recently gained attention in the soft robot field. Soft sensor calibration and characterization, as well as static and dynamic modeling and control of soft actuators, are two main categories of machine learning applications in soft robotics (Kim et al., 2021). However, the efficacy of these methods largely depends on the whether actuator and/or sensors used in the soft robot are soft and type them.

**FIGURE 4:**
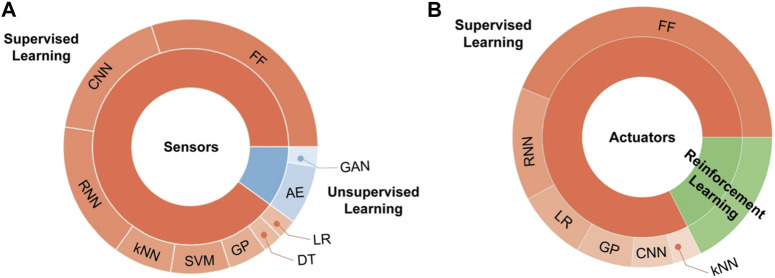
Learning techniques **(A)** soft sensors **(B)** soft actuators (Kim et al., 2021).

Using and calibrating soft sensors can be more challenging than rigid sensors. One effective solution to overcome these limitations is using learning-based methods. Machine learning methods can accurately characterize and calibrate the nonlinearity and hysteresis of soft sensors, which are difficult to express using analytical and experimental methods. Learning-based techniques can efficiently process enormous and counterintuitive datasets from several or array types of soft sensors, extracting valuable features and information necessary to complete tasks (Kim et al., 2021). For instance, resistive sensors and Recurrent Neural Network (RNN) methods have been used to obtain contact force and bending motion in soft actuators (Thuruthel et al., 2019). However, the nonlinear behavior of soft sensors can cause delays in estimating the configuration of the soft robot, even though they are more compatible with SPAs than rigid sensors. The application of artificial intelligence techniques in the soft sensor category depends on the function. For classification or object recognition, machine learning algorithms such as K-Nearest Neighbourhood (KNN) and Support Vector Machine (SVM) have been utilized, while RNN is frequently used for calibration of soft sensors. Convolutional Neural Network (CNN) is also an effective technique for soft robots with two-dimensional array data outputs, such as images (Kim et al., 2021). Figure 4A depicts the distribution of artificial intelligence algorithms employed in soft sensors based on a study by Kim et al. Other methods mentioned in both Figures 4A, B, including Gaussian Process (GP), Decision Tree (DT), Learning Rate (LR), Autoencoder (AE), Generative Adversarial Network (GAN), and Feedforward Neural Network (FNN), refer to other types of machine learning techniques (Kim et al., 2021).

Soft actuators are often integrated into the structure of rigid robots or soft robots. However, their hyper-elastic materials and resulting high degree of freedom can make the design of control system challenging (Case et al., 2015). Additionally, the time-varying characteristics of these materials can make it difficult to model soft actuators. To address these issues, there is a need to control high dimensions with fewer control inputs. Machine learning approaches have been employed for two purposes in this context: (1) modeling soft actuators with high degrees of freedom, and (2) controlling soft actuators with nonlinear behavior (Kim et al., 2021).

In other words, employing model-based control strategies for soft robots can be challenging due to the difficulty in obtaining the kinematic or dynamic model of the robot. Therefore, learning-based algorithms can be applied to obtain an analytical model for soft robots (Runge et al., 2017; Hyatt et al., 2019). For instance, FNN has been used to obtain the kinematic model of soft robots with soft actuators (Runge et al., 2017). Wiese et al. also used the Bayesian optimization for finding the appropriate hyperparameters (Wiese et al., 2019). Moreover, to overcome challenges such as high dimensionality in obtaining an inverse model, Rolf et al. proposed a learning-based technique (Rolf and Steil, 2013). Holsten et al. investigated a local model learning method to find the inverse kinematic of a soft robot (Holsten et al., 2019). Learning techniques can also play a major role in control strategies for soft actuators. Reinforcement algorithms like Q-learning have been shown to be useful for this purpose (Zhang et al., 2017). You et al. used a Q-learning technique to control a multi-segment soft manipulator (You et al., 2017). In (Satheeshbabu et al., 2019), an open-loop position control was implemented using deep Q-learning method. The use of Gaussian Process Regression (GPR) has been shown to be effective in controlling SPAs. For instance, in one study GPR was able to control a tripod soft mobile robot (Kim et al., 2019), while in another study, GPR was used to estimate the movement of SPAs (Fang et al., 2019). Figure 4B, based on Kim et al.'s work, illustrates the distribution of artificial intelligence algorithms used in soft actuators. Based on the figure, supervised learning methods such as FNN and RNN are the most frequently used methods in the soft actuator fields as well as the reinforcement learning. The main purpose of using machine learning methods in this field is controlling the robot specially its position (Kim et al., 2021). Selecting the suitable methods depends on the available information like numeric data from sensors or image from camera, etc. However, despite the potential of learning-based algorithms in modelling and controlling soft robots, there remain issues to be addressed, such as hysteresis in soft actuators and delay in soft sensors.

It is important to consider the control challenges associated with soft actuators when subjected to external forces or disturbances. While the use of sensors can improve their controllability, it is necessary to take into account the type of sensor used as it can affect the functionality of the robot and the feasibility of the manufacturing process (Molnar et al., 2018). Different methods for sensing exist, each with their own advantages and disadvantages (Molnar et al., 2018):

1) Feature and fiducial-based tracking: This sensing method includes electromagnetic (EM) sensing and visual servoing, which can be used to observe the deformation of soft robots. Although versatile and easy to use, occlusion and electromagnetic interface pose some drawbacks. It means that using electromagnetic interface in the presence of a metal hardware may lead to interference or noise and result in inaccurate tracking information. In the visual servoing also there is a possibility of the blocked view which may cause inaccuracy in the tracking process.
2) Mechanical sensors: Examples of these sensors include pressure sensors and cable encoders. They are accurate and easy to interpret, but require mechanical design considerations to measure the whole deformation changes of the soft robot.
3) Soft strain sensors: As previously mentioned, these sensors can be made from different materials. They are claimed to be robust and precise, but the fabrication process can be challenging.

Therefore, the addition of sensors to soft robots can enhance their controllability. As previously mentioned, due to material of the soft robot, it can be challenging to establish an accurate model of the robot and any discrepancies in the physical system of the robot and the model can result in different control outcomes. Therefore, in this case, open loop control is less likely to perform an accurate task. In other words, in terms of control, so as to have autonomous control and transcend open loop control, it is crucial to have sensory feedback. Sensors provide robots with perception which is vital to have intelligent and autonomous control. However, since the invention of the soft robotic field, unlike the progress in other fields from fabrication process to actuation systems, soft robot sensing still requires more investigation (Wang et al., 2018a). Moreover, integrating sensors into soft robots provides more information about position, force, strain, etc., for employing learning algorithms.

## 4 Control design of soft pneumatic robots

In the control design of soft pneumatic robots, the use of soft sensors and actuators has a significant impact on the control aspect of these robots. However, the presence of factors such as hysteresis, disturbance, noise, and backlash can make controlling soft robots a challenging task. One common method for controlling soft robots is through the use of calibration tests to obtain data on the required inputs and outputs for optimal performance, which can then be used as a control policy for the actuation system.

**FIGURE 5:**
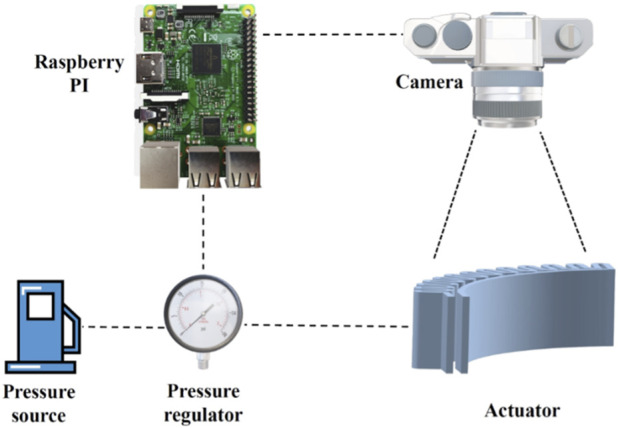
Experimental setup to control the soft actuator (Ji et al., 2020).

**FIGURE 6:**
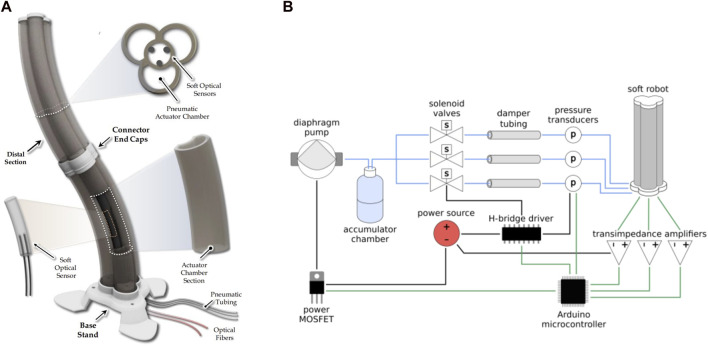
**(A)** Soft pneumatic robot with embedded soft optical sensor **(B)** controlling the pressure of the soft robot (Molnar et al., 2018).

Ji et al. proposed a closed-loop control for a 3D printed soft actuator using a Logitech C270 HD Web camera as visual feedback to observe the actual shape position of the soft actuator (Ji et al., 2020). The feedback is used to control the difference between the desired and actual positions using a PI controller and a low-pass filter, which calculates the amount of required pressure to inflate the soft actuators (Figure 5). However, the experimental test showed a small high-frequency vibration in the position control caused by elastic materials, errors from image processing methods, and instability in pressure inputs. Soft optical sensors also have been utilized to enhance the position control of a soft pneumatic robotic manipulator in the presence of external forces (Molnar et al., 2018). These sensors measure the strain of the soft robot based on light transmission between photodiodes and LEDs, which is used for position control. The robotic manipulator with embedded soft optical sensors is shown in Figure 6A. The position of the end effector is controlled by considering pressure and deformation alone or a combination of both. The results indicate that using both sensors improves controllability when the actuator is under external load. The control system is implemented using PID controllers with SMC VQ100 Series solenoid valves, and the method is implemented on an Arduino MEGA 2560, as shown in Figure 6B. Depending on the aim of the robot control, there is a demand for different types of feedback. In the work investigated by Ponraj, tactile sensor has been utilized to provide force feedback for closed-loop control on a soft pneumatic robot (Ponraj et al., 2017). In this study, the soft robot including one actuator for extension and one for bending has been employed for a cutting task. The feedback from tactile sensor determines the cutting action’s halting point. Sensor characterization has been done via Instron machine and Arduino microcontroller to discover the calibration equation.

**FIGURE 7:**
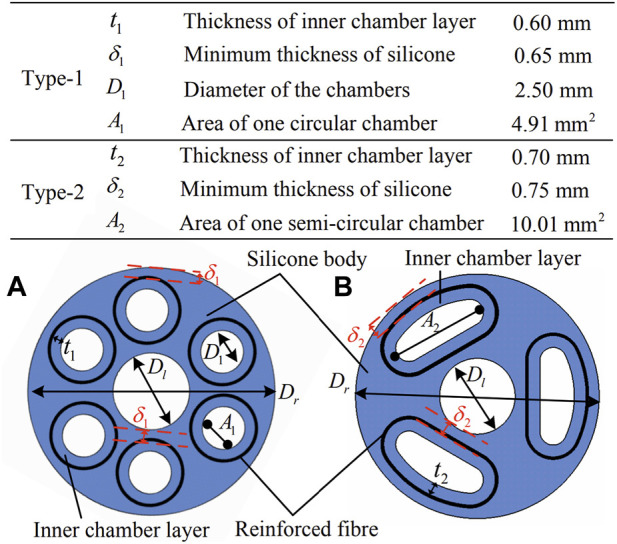
Cross-sectional for each actuator, **(A)** type-1: circular chambers, **(B)** type-2: semi-circular chambers (Shi et al., 2022).

From a control perspective, the mechanical design of the soft actuator significantly impacts its performance and controllability. Shi et al. explored two circular and semi-circular designs to measure and contrast the two models’ performance (Figure 7) (Shi et al., 2022). They assessed the actuators performance using two criteria, including the response-to-actuation ratio and hysteresis ratio. The study consisted of four experiments focused on bending and elongation, workspace, force generation, and stiffness. The experimental setup was as follows: an electromagnetic tracking system called NDI Aurora to measure the actuator configuration, the NI-DAQ USB-6341 connected to pressure regulators named Camozzi K8P, a compressor named BAMBI MD Range Model 150/500 and MATLAB software for collecting and processing data. The linear rail named Zaber X-LSM100A connected to the sensor named IIT-FT17 utilized to measure the force and stiffness. The authors defined response-to-actuation ratio and hysteresis ratio to measure the actuator performance for both circular and semi-circular models. Both actuators have been actuated and their response have been measured in four experiments with the same setup. In this study $A_{2}\approx {2A}_{1}.$ $A_{2}$ is the chamber area of the semi-circular actuator and $A_{1}$ is the chamber area of the circular actuator.

The response-to-actuation ratio parameter, denoted by β, has been defined as:

$$\beta =\frac{\Omega }{P},\;P=\frac{p_{1}+p_{2}+p_{3}}{3}$$ (6)

The parameter β , which represents the response-to-actuation ratio, is defined as the ratio of actuator response Ω to the generalized actuation pressure P. The generalized actuation pressure P is based on the three chambers in the circular actuator and three pairs of chambers in the semi-circular actuator. The actuator response is classified according to three parameters: the elongation-to-actuation ratio $\beta_{e}$ , the bending angle-to-actuation ratio $\beta_{b}$ , and the force-to-actuation ratio $\beta_{f}$ .

The hysteresis ratio, denoted as h, is defined as follows:

$$h=\frac{\left|\Omega_{f}-\Omega_{b}\right|}{\Omega_{m}}\times 100\%$$ (7)

The forward and backward responses of the actuation are denoted by $\Omega_{f}$ and $\Omega_{b}$ respectively, while $\Omega_{m}$ represents the maximum response. The authors found that smaller chambers facilitated the fabrication process, but required greater pressure to inflate. The results also indicate that the semi-circular actuator has superior performance in terms of bending and elongation, workspace, and generated force, while the circular actuator exhibits greater stiffness.

**FIGURE 8:**
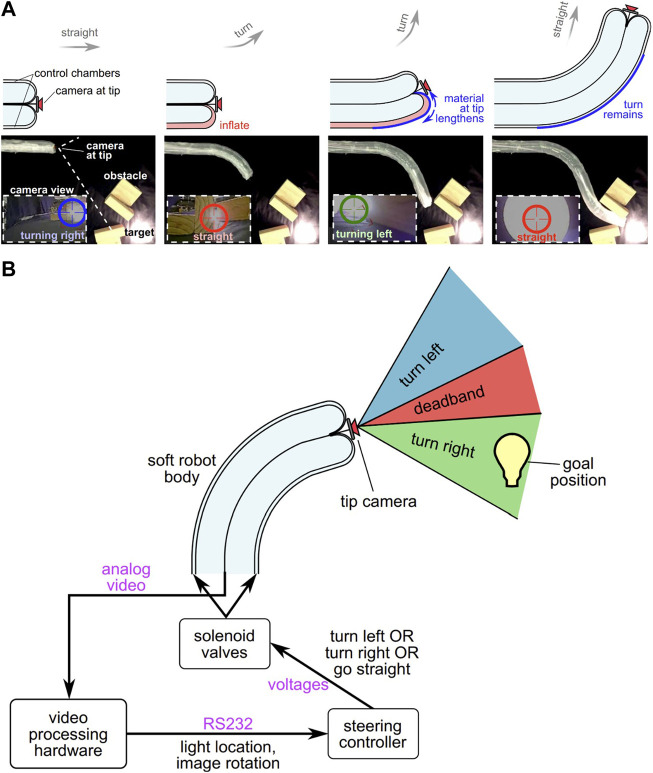
**(A)** Soft pneumatic robot with visual feedback **(B)** steering control system (Hawkes et al., 2017).

The soft pneumatic robot developed by E. Hawkes et al. is controlled based on the concept of growth (Hawkes et al., 2017). The robot, which everts from the tip, has two control chambers and a camera at the tip for visual feedback (as shown in Figure 8A). The steering control of the robot has been achieved by processing data from the camera and deciding which control chamber should be inflated to move toward the target while avoiding collisions with obstacles (as shown in Figure 8B). To calculate the location of the target and the rotation of the image, a video processing hardware called SLA-2000, developed by Sightline Applications Inc., has been used.

**FIGURE 9:**
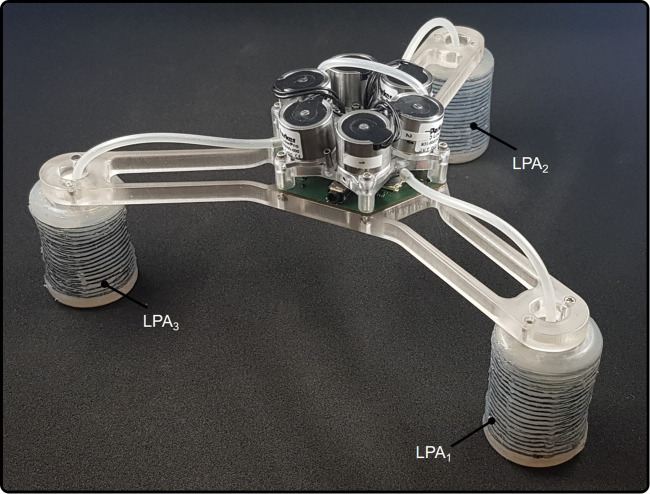
Three linear pneumatic actuators (LPAs) controlled by WCCIU (Manfredi and Cuschieri, 2019). ©2019 IEEE

The authors of (Manfredi and Cuschieri, 2019) proposed a Wireless Compact Control Unit (WICCU) for controlling pneumatic soft robots. The design includes proportional valves, pressure sensors, a Bluetooth RS232 serial module, and a digital signal processing (DSP) microcontroller. Closed-loop PID controllers were employed to regulate the pressure of the Linear Pneumatic Actuators (LPAs). Pressure sensors were used to measure the pressure in each chamber and to calculate the feedback error. Proportional valves were chosen over on-off valves due to their higher precision, as on-off valves require continuous activation, produce output spikes, and have limited precision. Output spikes can lead to resonance and instability of the system, making them unsuitable for precise control. Figure 9 shows the hardware setup used in this study.

**FIGURE 10:**
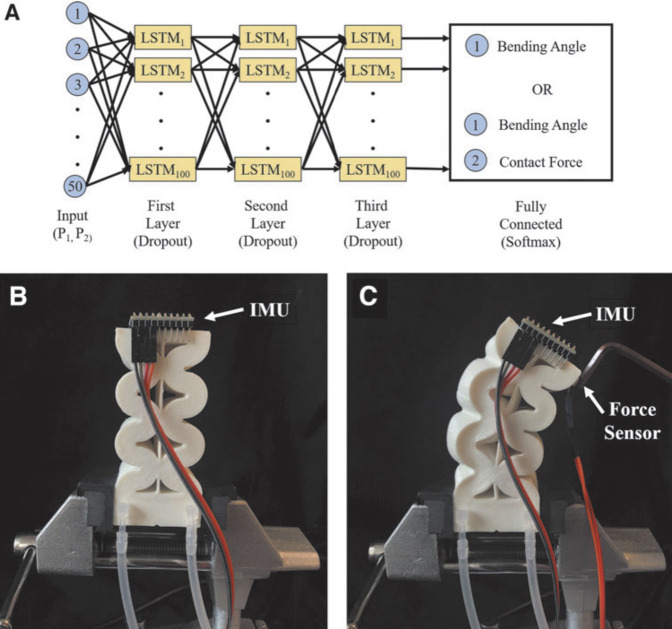
**(A)** Training process **(B)** IMU provides the ground truth for bending angle **(C)** force sensor provides the ground truth for contact force (Ang and Yeow, 2022).

As previously mentioned, soft robots can be controlled using artificial intelligent algorithms, and in a recent study by Ang and Yeow, a learning-based approach was proposed for a soft pneumatic actuator (Ang and Yeow, 2022). In this study, to obtain the ground truth for bending angle and contact force, an MPU-6050 Inertial Measurement Unit (IMU) and a Flexiforce A 1010 sensor were used respectively, along with pressure sensors to measure the pressure of each chamber. The Long Short-Term Memory (LSTM) method was employed to train the network to learn the pattern of changes in bending angle and contact force based on the applied pressure, and to estimate the shape of the actuator in two situations, with and without external contact. The actuator and attached sensors are shown in Figures 10B, C. The network was trained using pressure as the input and either bending angle or bending and contact force as the outputs, as shown in Figure 10A. However, the results indicated that there was an error in the predicted bending angle by the network for both with and without contact situations.

Fang et al. investigated another learning-based method utilizing local Gaussian Process Regression (GPR) to control a soft pneumatic robot (Fang et al., 2019). In this work, a camera has been mounted at the tip of the robot to provide visual feedback. The image frames captured from camera are processed via OpenCV to provide the displacement of the robot. Via local GPR, an inverse mapping between the robot motion provided by camera and actuator space has been learned. Based on the paper, the rationale behind localizing GPR is boosting the computational efficiency and decreasing the input matrix dimensions. K-means clustering algorithm has been utilized for this purpose.

Due to the capabilities of reinforcement learning methods, there have been recent investigations utilising them more frequently. In these methodologies the main goal is ascertaining the control policy rather than finding the model of the robot. Centurelli et al. explored a method based on deep reinforcement learning named Trust Region Policy Optimization (TRPO) to control a soft pneumatic manipulator (Centurelli et al., 2022). In this work, approximated the forward dynamic model of the robot using a LSTM network, and then implemented a deep reinforcement learning technique based on this approximation to control the robot. The experimental setup was as follows: The VICON motion capture system, MATLAB software, an Arduino Due microcontroller and pressure regulators.

Satheeshbabu et al. presented an open loop control via deep reinforcement learning to control the position of a soft pneumatic continuum arm (Satheeshbabu et al., 2019). The combination of Markov Decision Process (MDP) and Deep Q-Learning (DQN) has been employed to design the control strategies for this robot. The experimental setup to validate the method was as follows: a pneumatic pressure source connected to a pressure regulator named SMCITV0050-2NU, LabVIEW along with myRIO-1900 and a 3D digitizer from MicroScribe. The methodology validation has been done in both simulation and the continuum robot prototype indicating better performance for simulation than robot prototype.

You et al. proposed a multi-segment pneumatic manipulator using reinforcement learning as well (You et al., 2017). In this study, Q-learning method was utilised to control the robot and the suitable control signal was sent to the pressure regulator to actuate the robot. They validated the methodology using their robot prototype indicating high precision and robustness.

### 4.1 Control design of soft pneumatic endoscopic robots

In this section, we will discuss previous works on soft pneumatic actuators specifically designed for colonoscopy applications. Naghibi et al. developed a soft robot with multi-level stiffness for endoscopic procedures (Naghibi et al., 2019). The robot used chambers for both actuation and stiffness purposes. By utilizing coffee powder inside the chambers based on the granular jamming principle, researchers defined three different stiffness levels: no stiffness (actuation only), level one stiffness (actuation and vacuuming of the opposite chamber), and level two stiffness (actuation and vacuuming of two adjacent chambers). The purpose of this design was to provide sufficient force for surgical intervention in soft endoscopic robots where stability and applied force are often inadequate (Laschi et al., 2017). To control the bending angle (*θ*) and rotation (*∅*), characterization data based on a calibration method was used, where the pressure needed to inflate each chamber was measured to achieve any desired *θ* and *∅*. Additionally, haptic feedback was obtained using a PHANTOM Omni for force estimation.

**FIGURE 11:**
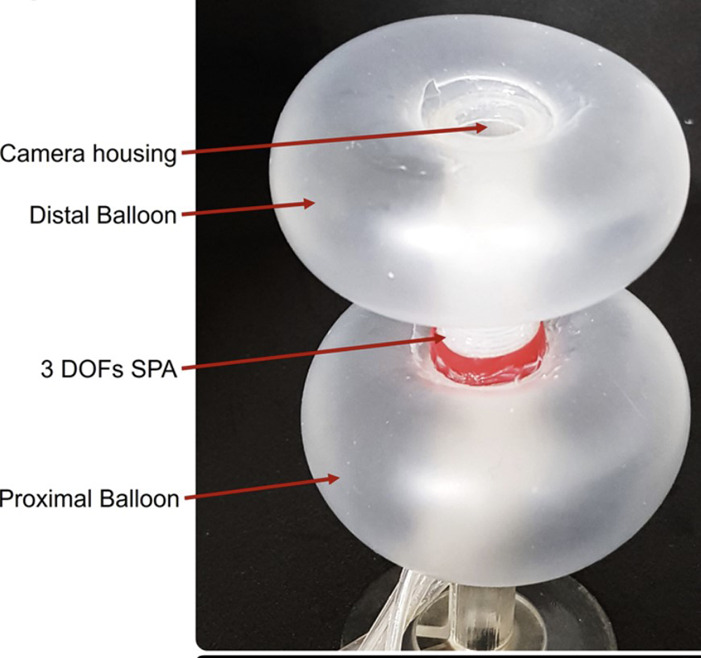
Spid design (Manfredi et al., 2019).

Manfredi et al. utilized a SPA to create a Soft Pneumatic Inchworm Double Balloon (SPID) for colonoscopy purposes (Manfredi et al., 2019). The SPID provides three degrees of freedom, one for extension and two for rotation around two axes. The robot’s locomotion process involves activating the proximal balloon, followed by activating the SPA and the distal balloon, then deactivating the proximal balloon and finally the SPA. This approach has multiple benefits, including providing greater contact force against the colonic wall while reducing pressure on it, allowing for precise control and high manoeuvrability due to the increased DOFs, enabling navigation through tight corners and bends in the colon, improving stability by allowing the robot to maintain its position within the colon and providing ample internal space for accommodating necessary apparatus such as camera, cable for colonoscopy procedure. Figure 11 depicts the SPID design.

**FIGURE 12:**
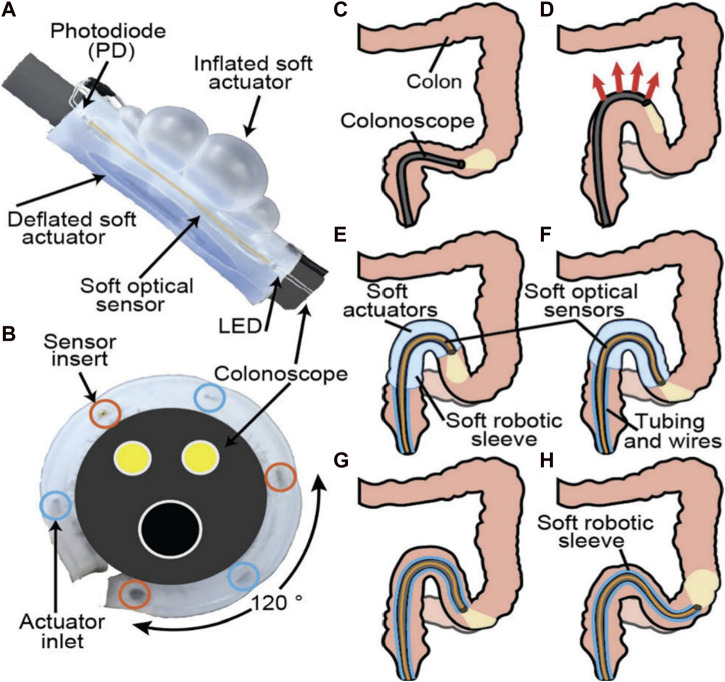
Soft robotic sleeve wrapped around the colonoscope and its functionality during the colonoscopy (McCandless et al., 2021).

A soft robotic sleeve is another proposed method for the colonoscopy procedure, as described in a study by McCandless et al. (2021). This approach involves covering the endoscopic instrument with a disposable soft sleeve that serves as an “add-on” device. The proposed soft robotic sleeve is shown in Figure 12. Soft optical sensors are embedded in the sleeve to monitor any bending or shape alterations based on changes in light loss. By using characterization data obtained from sensor calibration tests, the force applied to the colon can be estimated. If the applied force exceeds a predefined threshold, the soft sleeve is inflated to distribute the contact force over a larger area. This control method helps to reduce the pressure applied to the colon wall and improve the overall safety and efficacy of the colonoscopy procedure.

From a control standpoint, accurate feedback is crucial in closed-loop control, especially when the robot interacts with the human body, such as in endoscopy procedures. Three common methods used for controlling the position and orientation of a robot are model-based control (kinematics), electromagnetic (EM) tracking, and image-based tracking. However, model-based control is not useful for flexible robots due to the lack of accurate models, while EM trackers have limited use in medical applications due to interference from electromagnetic interfaces and metallic tools. Medical imaging modalities are used to obtain feedback before and during the operation, and applying image processing methods to these images can provide the robot’s control system with efficient position feedback (Azizian et al., 2014). The term “visual servoing” is often used when controlling a robot with visual feedback (Hutchinson et al., 1996). In image-based tracking, real-time images must be obtained quickly to prevent aliasing, a significant factor in visual servoing control for medical robots (Azizian et al., 2014).

However, utilizing visual servoing in medical robots poses significant challenges (Azizian et al., 2014). These challenges include:

1) Reliable and robust image-based detection and tracking methods.
2) Image resolution and tracking accuracy are interdependent, with higher image resolution leading to more accurate tracking.
3) The incidence of image capture affects the control system.
4) Delay resulting from image capture, transfer, and processing can affect the control loop, with high delay leading to instability.
5) Efficient image processing techniques that are computationally feasible.
6) Offline calibration and registration can improve efficiency.

**FIGURE 13:**
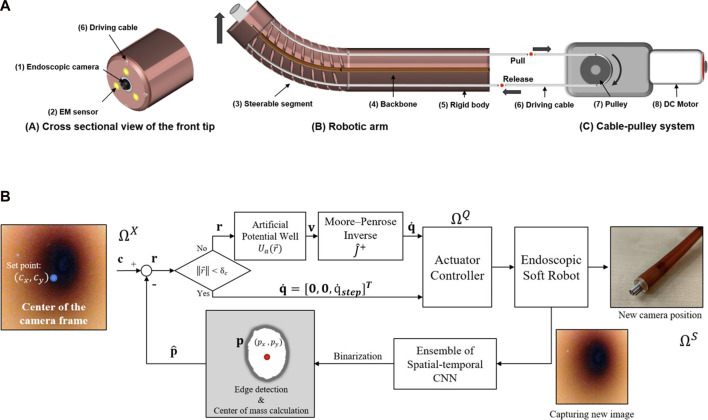
**(A)** Mechanism of the endoscopic soft robot **(B)** Model-less control using visual servoing (Lazo et al., 2022).

Despite these challenges, visual servoing has been successfully utilized in autonomous intraluminal navigation of a soft endoscopic robot in medical applications (Lazo et al., 2022). This robot has three degrees of freedom actuated by cables (Figure 13A). The robot is moved inside the lumen by detecting the center of the lumen using the CNN method. Visual servoing is then used to approximate the image Jacobian, which maps the robot’s actuation space and task space in the image. The aim of the Jacobian is to keep the robot position at the detected center and move it forward at a constant pace. Artificial Potential Well is proposed to achieve this control demand, which uses two PID and one P controllers for two DC motors and one linear stage, respectively. The proposed control strategy for the soft robot is demonstrated in Figure 13B.

Trovato et al. employed reinforcement learning methods to control a different robot design for colonoscopy procedure (Trovato et al., 2010). The design of this robot consisted of a front body providing clockwise rotation and a rear body providing anticlockwise rotation connected via a DC motor. This design, based on the screw-like locomotion, helps the robot move forward and backward inside the colon. However, the velocity and direction of the movement were determined using the Q-learning and State-Action-Reward-State-Action (SARSA) algorithms. According to this paper, navigating the robot in tight passage of the colon is akin to the Mountain Car problem which can be addressed using Q-learning and SARSA. Nevertheless, movement in the bending area of the colon remains a challenge.

## 5 Conclusion

In this paper, we have reviewed potential solutions for the control of soft endorobots in the context of colonoscopy with its design and control challenges. There are several methods to power and actuate an endoscopic robot with their own merits and demerits. The selection of these methods depends on the design requirements, particularly the actuation space. Achieving autonomous and intelligent control requires feedback, thus, sensing elements have a profound influence on the robot control and sensor selection depends on the suitable parameter needed to be monitored in the control loop. Feature and fiducial-based tracking, mechanical and soft sensors are prevalent sensing methods in this area. In addition to the impact of the sensing and actuating system on the control, soft endoscopic robot can be controlled based on the model of the robot or without a model. Due to the characterization of soft robots such as high degree of freedom and nonlinearity, accurately modelling them may not be feasible. This may require model-free control approaches based on learning methods, as opposed to model-based control using an exact system model. However, utilizing machine learning algorithms in this field can be used for both finding the model and/or controlling the robot. In the case of finding the robot model, the relationship between inputs and outputs of the robot is estimated while in the case of control, the focus is on finding the suitable control signal. Some parameters like hysteresis and delay, resulting from soft actuator and soft sensors respectively, may lead to instability of the robot and make the control task more demanding.

However, available information about system and its environment as well as control demands such as the amount of required accuracy, stability and robustness have a profound influence on the control strategy as well. Soft robots can also be controlled using characterization data collected from calibration tests, but the control system may not be suitable in all dynamic and unknown environments.

In conclusion, the type of sensors and actuators used in the robot, having prior knowledge of the environment, the type of the feedback and the design of the robot play a prominent role in the control strategy selection. However, machine learning algorithms demonstrate the capability to learn not only the relationship between input and output for finding the model but also the control policy, making them appear more likely to succeed in this area.

## Footnotes

1 Prismatic-Revolute-prismatic.
